# Although myeloid‐cell RGS2 knockout does not affect Ang II–induced hypertension/organ damage in mice, high RGS2 in Gitelman/Bartter syndromes—human models of endogenous Ang II signaling antagonism—is associated with hypotensive/antiremodeling effects

**DOI:** 10.14814/phy2.71110

**Published:** 2026-09-21

**Authors:** Lorenzo A. Calò, Martina Cacciapuoti, Paul A. Davis

**Affiliations:** ^1^ Department of Medicine, Nephrology University of Padova Padova Italy; ^2^ Department of Nutrition University of California Davis California USA

Myeloid cells are believed to contribute to hypertension by driving vascular inflammation, promoting T‐cell activation, and regulating renal sodium retention (Hunter & Harrison, 2026). Yet Regulator of G‐protein signaling 2 (RGS2) is highly expressed in myeloid cells, presenting a potential paradox given RGS2's negative regulatory activity on angiotensin (Ang) II signaling via Ang II type 1 receptors (AT1R) and their established role in hypertension and cardiovascular‐renal remodeling (Heximer et al., 2003; Semplicini et al., 2006; Zhang et al., 2011). To probe this, Nakagawa and coworkers (Nakagawa et al., 2026) developed an elegant conditional myeloid‐specific RGS2 knockout mouse model (RGS2 LysM‐KO). Using this model, they documented the effects of chronic Ang II treatment, which, contrary to their own and general expectations, did not show any significant differences in blood pressure, cardiac and renal damage, vascular dysfunction, or inflammatory profiles in response to Ang II. These results led the authors to conclude that myeloid RGS2 is not involved in Ang II‐induced hypertension or cardiovascular‐renal organ damage (Nakagawa et al., 2026) and suggest that the severe, damaging hypertension seen in whole‐body RGS2 deficiency is driven exclusively by altered RGS2 signaling in vascular and renal epithelial tissues, not the immune system.

Given these results, we would like to offer some insights into RGS2 signaling that we have obtained using a human model of endogenous Ang II signaling antagonism, i.e., the mirror image of hypertension, Gitelman's and Bartter's syndromes (Calò et al., 2014). Gitelman's and Bartter's syndrome are rare genetic tubulopathies caused by gene defects in specific kidney transporters and ion channels (Ravarotto et al., 2022). These conditions are characterized by activation of the renin‐angiotensin‐aldosterone system, with high Ang II and aldosterone levels; however, despite this, patients with GS/BS present with hypotension or normotension rather than hypertension. In addition, Gitelman's and Bartter's patients show hyporesponsiveness to pressors and activation of antiatherosclerotic and antiremodeling defenses, including reduced Rho kinase signaling, increased nitric oxide, and reduced oxidative stress and oxidative stress‐related signaling, despite high Ang II levels (Calò et al., 2014). Of particular note, we have shown that negative regulation of Ang II‐mediated AT1R signaling by RGS2 includes, in these patients, attenuation of Ang II‐induced short‐ and long‐term signaling (intracellular Ca^++^ release and Ca^++^‐PKC signaling in vascular smooth muscle cells and Ang II proliferative and profibrotic responses, respectively) (Calò et al., 2014). Notably, in fibroblasts from Gitelman's and Bartter's patients, RGS2 RNA and protein abundance are increased compared with healthy normotensive subjects (Calò et al., 2004; Calò et al., 2014), and silencing RGS2 in fibroblasts from Gitelman's and Bartter's patients restored a hypertensive‐like Ang II response (increased intracellular Ca^++^ release and ERK 1/2 phosphorylation) (Calò et al., 2008; Calò et al., 2014). These results, combined with those of Nakagawa and coworkers, demonstrate that RGS2 regulation of Ang II signaling via Gq/AT1R and the induction of hypertension and cardiovascular‐renal remodeling differ substantially by the level and location of the RGS2 system probed. We look forward to Nakagawa and coworkers' future work addressing the major limitations they noted in their study, including Ang II dose and time points, as well as more selective targeting methods for myeloid‐specific ablation, which might provide further important information.

## AUTHOR CONTRIBUTIONS

**Lorenzo A. Calò:** Conceptualization; investigation; supervision. **Martina Cacciapuoti:** Investigation. **Paul A. Davis:** Conceptualization; investigation.

## CONFLICT OF INTEREST STATEMENT

The authors have no conflict of interest.

## ETHICS STATEMENT

None.

## LINKED ARTICLES

This article is linked to Nakagawa et al. papers. To view this article, visit https://doi.org/10.14814/phy2.71023.
